# On the role of demagnetizing tensors in arbitrary orientations of general ellipsoids and implications for MRI safety assessment

**DOI:** 10.1002/mp.70444

**Published:** 2026-04-19

**Authors:** Tomppa Pakarinen

**Affiliations:** ^1^ Faculty of Medicine and Health Technology Tampere University Tampere Finland

**Keywords:** demagnetizing tensors, demagnetizing factors, magnetization, magnetic fields, MRI safety

## Abstract

**Background:**

Demagnetizing tensors describe the shape anisotropic contribution of magnetic susceptibility, that is, how an object's shape and orientation affect its internal magnetization. Ellipsoids possess a unique geometric property by exhibiting homogeneous internal magnetization, enabling a purely geometrical characterization. A general description of the demagnetizing tensor under arbitrary rotations would broaden their applicability across various fields, most notably in magnetic resonance safety evaluation.

**Purpose:**

Demagnetizing tensor is a well‐defined concept in the ellipsoids principal frame, though its transformation under three‐dimensional reorientation is often overlooked, a justifiable omission for spheroidal solutions of the Poisson equation. However, this does not hold for general ellipsoids under arbitrary reorientation. This work is motivated by the concerns in magnetic resonance imaging safety and its practical evaluation in clinical environments, aiming to extend and simplify the process.

**Methods:**

This work demonstrates the validity of directly rotating the orthogonal basis solutions, derived from Poisson's equation, and uses the procedure to evaluate a practical approximation, based on orthogonal area‐projections. The approach is also applied to generalize force and torque calculations for ellipsoids under three‐dimensional reorientation.

**Results:**

The method shows an exact match with the numerical solution and agrees with the standard scalar expressions for translation force and torque. Additionally, a unique connection to the MRI magic angle is found as the point of convergence for prolate spheroid aspect ratios under rotation. The area‐projection approximation was demonstrated to perform fairly across prolate and oblate spheroids. Similar approximations might extend to irregular shapes, but numerical approaches remain preferable due to the complexity of internal field distributions.

**Conclusion:**

The presented approach offers an alternative method for force and torque calculations, such as those provided by ASTM, also generalizing the conventional approach. The area‐projection approximation via SVD offers a straightforward extension to the original method. Finally, the connection between demagnetizing tensor rotation and the magic angle provides a new perspective on the phenomenon.

## 1 Introduction

Magnetization of an object, subject to an external magnetic field depends primarily on the material's magnetic susceptibility (χ), geometry and orientation of an object with respect to the external field. The induced magnetization (M) gives rise to demagnetizing field $(H_{d}$), opposing M. In anisotropic geometries, the demagnetizing field is found via magnetostatic equations by solving the magnetic scalar potential (φ), resulting from the internal magnetization. The usual path includes solving the Poisson equation for magnetic scalar potential

(1) $$\nabla^{2}\varphi \left(r\right)=\left\{\begin{array}{c} \nabla \cdot M\left(r\right)=-\nabla \cdot H_{d}\left(r\right)=4\pi \rho_{c}\\ 0 \end{array}\right.$$

where $\rho_{c}$ is the charge density. The top part of (1) holds within the specimen, and the zero‐condition holds outside the specimen. To simplify, a uniform magnetization (∇·M=0) is often assumed, reducing the problem to Laplace's equation. The validity of the assumption depends largely on the inspected shape, material anisotropy and on the application at hand. Though an important peculiarity of ellipsoidal geometries is that such definition applies in general. In addition, the solution is typically sought only for domains without free charge carriers. This is a fundamental condition for the demagnetizing field relation

(2) $$H_{d}\left(r\right)=-\nabla \varphi \left(r\right)$$

to be consistent with Ampere's law.^1^ In the general case, Poisson equation is solved over the volume and at the surface of the object using Green's functions, the solutions for the Laplacian operator in these domains

(3) $$\varphi \left(r\right)=\frac{1}{4\pi }∰\frac{\nabla^{\prime }\cdot M}{|r-r^{\prime }|}dV^{\prime }+\frac{1}{4\pi }∯\frac{n\cdot M}{|r-r^{\prime }|}dS$$

Due to uniformity assumption that is, zero‐divergence within the specimen, the volume integral term vanishes, leaving the surface integral as the only contributing factor to the scalar potential. The linear relation between $H_{d}$ and the corresponding magnetization is described by rank‐2 demagnetizing tensor ($N_{ij}$)

(4) $$H_{in}\left(r\right)=H_{0}-H_{d}=H_{0}-N_{ij}M,$$

where $H_{d}$ and M are now reduced to vectors, $H_{0}$ is the applied field. The principal axes of the demagnetizing tensor are referred to as demagnetizing factors $N_{ii}=\left\{N_{xx},N_{yy},N_{zz}\right\}$, for which the first principal tensor invariant holds via

(5) $$\sum_{i}N_{ii}=1.$$

The demagnetizing tensor is found by solving the surface integral in (3) and via the relations in (2) and (4). Computation is in most cases carried out numerically, since general solutions even for primitive shapes, are typically incomplete. Therefore, only few regular geometries exhibit spatially constant magnetization, rendering the problem difficult for analytical solutions. Regardless, it is common to approximate the net or average demagnetizing contribution in primitive shapes using the uniformity assumption,^2^, ^3^, ^4^, ^5^ introducing some alternative approaches.

### 1.1 Motivation and clinical applications

The motivation for this work and its potential applications are in medical magnetic resonance imaging, where heating effects, translational forces and torque on foreign bodies and implants are a fundamental concern.^6^, ^7^, ^8^ The work focuses on the demagnetizing tensor and its properties in static magnetic fields, hence considering only force and torque. In clinical practice, each patient case must be evaluated individually. In majority of clinics, patient specific simulations are not accessible or feasible, and often a swift decision is based solely on empirical judgment. Construction of approachable methodologies with sufficient accuracy could support decision making or at least provide intuitive insight into the governing physical phenomena. Ideally, the demagnetizing tensor can be directly applied to estimate the total forces exerted on the specimen.

### 1.2 Previous work and aim

Practical approaches to approximate demagnetizing tensors on primitive geometries have been investigated extensively for decades. Past research has been focusing primarily on the orthogonal orientations of the specimen with respect to the external magnetic field lines, also reducing the inspection to the diagonal demagnetizing factors.^2^, ^9^, ^10^, ^11^ The analytical groundwork for spheroidal bodies in the orthogonal orientations was first introduced by Osborn in 1945.^12^ The solutions for individual aspect ratios have been later revisited by several groups, most recently by Etse and Mininger in 2022, using an iterative approach in several geometries. In the same year, Kiss & Bakonyi approached the problem through graphical approximations. ^11^, ^13^ Earlier, Pugh et al. (2011) had also presented precise reevaluation of demagnetizing factors for various geometries through finite element modeling (FEM).^10^ Complete analytical formulations are often limited to exceptions, as exact solutions rarely exist, and the divergence of internal magnetization in (3) cannot be ignored for most geometries. Consequently, practical approaches rely on numerical simulations in solving the scalar potential or, on approximations that allow for sufficient evaluation of the net demagnetizing field in a given application.^14^ One such approach, also included in this work, is based on area projection approximation presented by Bahl et al., in 2021.^9^ In their approach, the surface integral over the normal vector in (3) is reduced to simplified area projections, being computationally efficient and conceptually intuitive. In general, simplified expressions are relevant, as they are approachable to a broader occupational audience, and straightforward to implement even with basic computational tools.

Whereas orthogonal approximations have been largely investigated for several primitive geometries, arbitrary object rotation and its effect on the demagnetizing field remains as a less studied concept. Solutions under 3D‐rotations are not necessarily trivial, either for approximations or exact solutions, yet no prior inquiries within MRI research have been published, to the best of my knowledge. Single axis trigonometric methods for rotationally symmetric objects have been previously introduced for magneto mechanic applications by Jackson et al. in 2006 and Abbott et al. in 2007.^15^, ^16^ Similar approaches with corresponding physical test setups have been recommended by regulatory bodies such as ASTM and IEC in their magnetic resonance imaging safety standards.^17^, ^18^

The closest equivalents for the presented problem have been investigated by Moskowitz and Torre in 1967 through analysis of the theoretical aspects of demagnetizing tensors, including rotational properties. Similar approaches have been utilized more recently in micro magnetic control applications and quantum magneto mechanics by Bort Soldevila et al. in 2024.^19^, ^20^

In this work, an approximative evaluation is implemented using the area‐projection method and, as such, also serves as a potential extension to the procedure introduced in.^9^ Moreover, the proposed direct tensor rotation offers an approach to reformulate the orientational effects on translational forces and torque for the traditional methods for example, by Abbot et al. and ASTM. The approach presented is also conceptually aligned with applications in adjacent fields. ^16^, ^19^, ^20^ With successful implementation, the method generalizes the standard single axis expression for spheroids to general ellipsoids under arbitrary 3D‐rotations, conveniently contained in a single tensor.

## 2 Methods

### 2.1 Translational force

The translational force ($F_{Trans}$) exerted on magnetizable bodies can be calculated as the gradient of the inner product between magnetization and external field vectors

(6) $$F_{trans}=\nabla \left(B\cdot M\right),$$

from which it is apparent that $F_{trans}$ is zero for the homogenous field region. The contribution of susceptibility and scalar demagnetizing factors can be straightforwardly derived by substituting (4) and $M=\chi H_{in}$ to (6)

(7) $$F_{trans}=\frac{F_{g}}{\mu_{0}g\rho_{m}}\;\frac{B_{0}}{\left(\frac{1}{\chi }+N_{k}\right)}\left|\nabla B\right|,$$

where $F_{g}$ is the gravitational force and $\rho_{m}$ is the mass density.^21^ Consequently, in ferromagnetic materials with high susceptibility, $F_{trans}$ scales primarily with reciprocal of $N_{k}$. However, ( 7) inherently assumes orthogonal alignment with the magnetic field lines. A trigonometric extension defines exerted translational force for spheroids under planar rotation as

(8) $$F_{trans}=\frac{V}{\mu_{0}}\left(\frac{\cos^{2}\theta }{\left(\frac{1}{\chi }+N_{n}\right)}+\frac{\sin^{2}\theta }{\left(\frac{1}{\chi }+N_{⊥}\right)}\right)B_{0}\left|\nabla B\right|,$$

where θ is referred as the deflection angle, $N_{⊥}$ is the demagnetizing factor perpendicular to $N_{n}$ and within the deflection plane, and V is the object volume.^22^ The expression, however, considers single axis rotation through scalars $N_{n}$ and $N_{⊥}$ and hence, $N_{ij}$ as a tensor quantity is not explicitly addressed. Such approaches are typical also in the related research,^7^, ^15^, ^16^ which is fair for spheroids due to rotational object symmetry. However, this does not extend to general ellipsoids with three unique axes of symmetry.

It should be noted that at full magnetic saturation in very high magnetic field strengths, the practical need to compute $N_{ij}$ diminishes due to its negligible effect on translational forces at magnetization saturation asymptote. That is, when the external field strength has completely overcome the demagnetizing effects.^21^ Though shape anisotropy is still relevant in fringe fields and in modern low‐field imaging systems, the omission is often considered as valid for a high field MRI scanners near the bore.

### 2.2 Torque

Whereas the risks associated with translational forces consider mainly objects external to the human body, implant and foreign object torsion is a relevant risk to the nearby structures, such as arteries and neural tissue. In addition, torque even without relevant torsion tends to compromise patient comfort during the imaging procedure. Torque reaches its peak value at the highest field strength within the homogenous region of $B_{0}$. The fundamental relation between exerted torque and a magnetic dipole (***m***) is

(9) T=m×B.

There is a prominent body of research around the topic, including the definitions provided by International Standards Organization (ISO) and additional recommendations for torque measurements by ASTM. ^7^, ^16^, ^18^, ^21^ A typical formulation of the torque for a single axis rotation in the linear region is

(10) $$T_{n\times ⊥}=\mu_{0}\;V\frac{\left|N_{n}-N_{⊥}\right|}{2N_{n}N_{⊥}}{\left|H_{0}\right|}^{2}\sin2\theta ,$$

where torque is dependent on $H_{0}$ up to the saturation field strength ($H_{sat})$, that is, where magnetization saturation ($|M_{s}|$) has been reached. However, the linear depiction is typically not sufficient in describing torque accurately inside the MRI bore, especially when the $B_{0}$ field strength exceeds the clinically conventional 1.5 T.^22^ In these field strengths, magnetic saturation has been reached in most soft magnetic materials, and nonlinear coupling between field strength and magnetization vector direction α starts to dominate.^16^, ^22^, ^23^ Nonlinear behavior is typically derived at saturation threshold by finding the roots for α through the scalar form of magnetic energy minimization

(11) $$\frac{dE_{T}}{d\alpha }=\frac{{\left|M_{s}\right|}^{2}}{2\mu_{0}}\left(N_{n}-N_{t}\right)\sin\left(2\alpha \right)-\left|M_{s}\right|\left|H_{0}\right|\sin\left(\theta +\alpha \right)=0$$

leading to the first term in (12) and corresponding, for instance, to in the ASTM standard test method designation.^17^

(12) $$\left|T\right|=V\left|M_{s}\right|\left|H_{0}\right|\cos\left(\theta +\alpha \right)=\frac{\mu_{0}V\left|N_{n}-N_{⊥}\right|}{2}{\left|M_{s}\right|}^{2}\sin\left(2\alpha \right),$$

where the latter term corresponds to the expression in.^16^

### 2.3 Direct tensor rotation

Considering the dependence of demagnetizing tensors solely on geometrical properties, that is, aspect ratio and orientation with respect to the external magnetic field, it may be intuitively compelling to directly rotate the orthogonal tensor instead of the shape.

(13) $$N_{ij}^{\prime }=RN_{ij}R^{T}|R\in SO\left(3\right),$$

**FIGURE 1:**
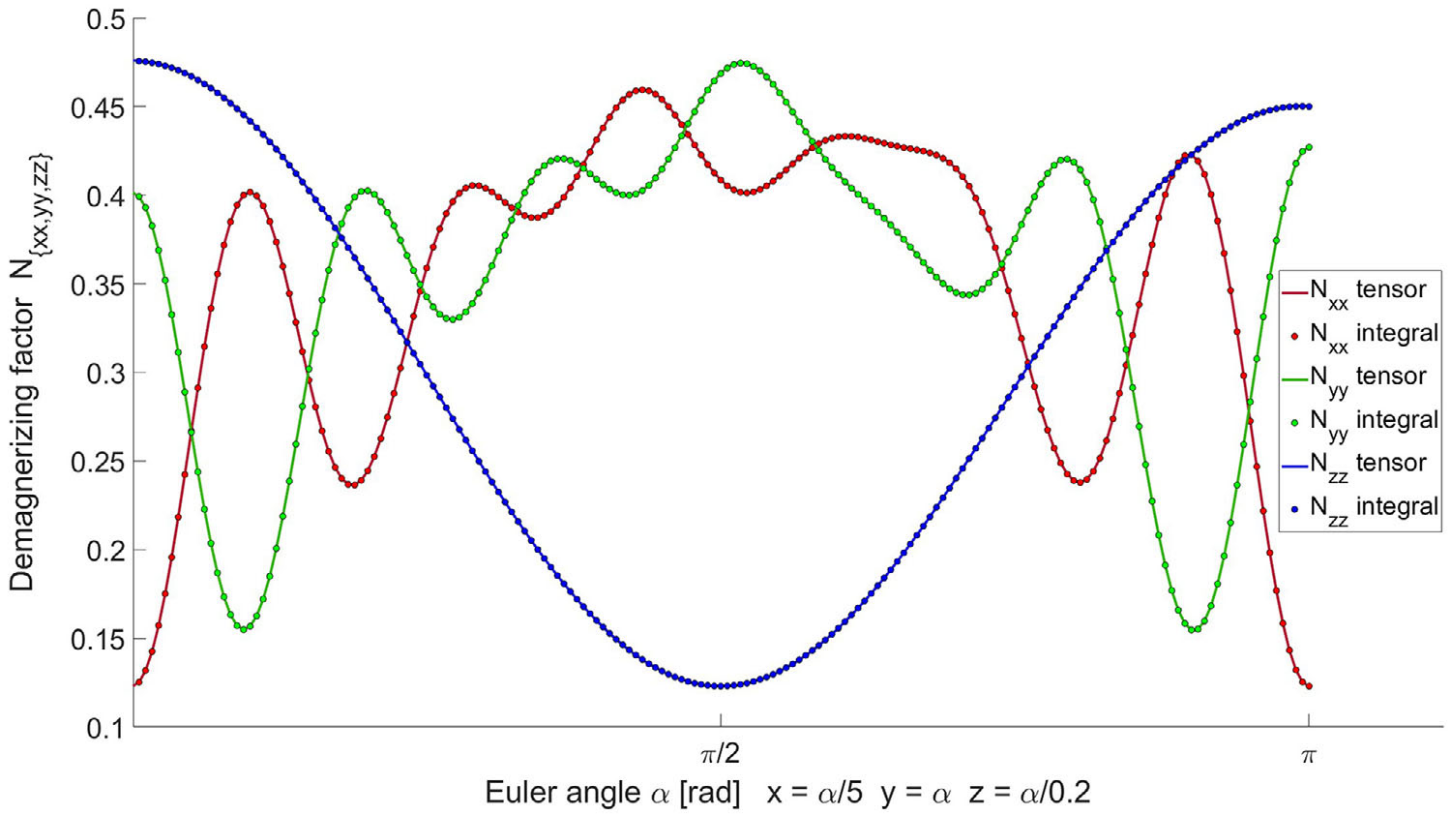
Diagonal demagnetizing factors for a general ellipsoid (a/b=0.4,c/a=0.342) via numerical surface integration of (3) after rotation and directly rotating the orthogonal basis solution for the demagnetizing tensor.^12^ ^.^

Thus, sparing the extra work in solving the Poisson equation after the rotation. Indeed, such intuition does apply to the exact solutions. A quantitative comparison between numerically solved Poisson equation and the direct tensor rotation for the diagonal terms are presented in Figure 1.

The comparison in Figure 1 is presented as a trivial case, limited only by sampling and computational accuracy, as expected. Repeating the computation for the tabulated basis solutions in ^12^ with randomized rotations are equivalent, without exceptions. $N^{\prime }$ in (13) can be straightforwardly applied to the general expression of translational force in (6) and torque in (9) in the linear region, yielding the tensor forms as

(14) $${F^{\prime }}_{trans}=\frac{V}{\mu_{0}}\left\{{\left(I+\chi N^{\prime }\right)}^{-1}\chi \right\}H_{0}\left|\nabla B\right|,$$

and for torque

(15)

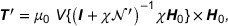

where I is the identity matrix and $|H_{0}|$ ranges from zero until $H_{sat}$. At magnetization saturation, solving the energy minimization problem in (11) reads in the tensor form as

(16) $$\min\left\{\frac{1}{2}M_{s}^{T}N^{\prime }M_{s}-M_{s}\cdot H_{0}\right\}$$

where 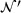 is rotated with (13) and direction of $M_{s}$ is solved, which can then be used to calculate the torque directly.

### 2.4 Area projection approximation

When the inspected geometry fulfills or closely approximates conditions {*c*_1_, *c*_2_, *c*_3_}, the average demagnetizing factors can be estimated by

(17)

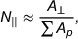

where $A_{⊥}$ is the surface projection area in the perpendicular plane to the magnetic field direction and $N_{\left|\right|}$ is the scalar demagnetizing factor in the direction of $H_{0}$, and $A_{P}$ corresponds to each orthogonal projection on a static basis.^9^ Conditions $c_{1-3}$ are defined here as

(18) $$\left\{\begin{array}{c} c_{1}:\;The\;surface\;is\;whole\;and\;continuous\\ c_{2}:\;The\;surface\;geometry\;is\;convex\\ c_{3}:\;Geometry\;has\;axial\;symmetry \end{array}\right.$$

The spatial variance of material susceptibility is assumed to have negligible effect on the overall magnetization,^9^, ^14^ allowing a solely geometrical characterization. However, directly rotating the orthogonal solutions is not applicable, since the constructed diagonal matrix is not an actual tensor and would also imply equivalence of transforms prior ($R^{3}\to R^{3}$), versus after projection ($R^{2}\to R^{3}$). Hence, demagnetizing factors for a given geometry must be evaluated via the orthogonal premise through $A_{P}$ on the static magnetic field coordinates after the reorientation.

For a vector space V⊂R ^3^ , an orthogonal projection is defined as P:V→V and $P^{2}=P=P^{T},s.t.$

(19) $$P_{xy}=\left[\begin{array}{ccc} 1 & 0 & 0\\ 0 & 1 & 0\\ 0 & 0 & 0 \end{array}\right],P_{xz}=\left[\begin{array}{ccc} 1 & 0 & 0\\ 0 & 0 & 0\\ 0 & 0 & 1 \end{array}\right],P_{yz}=\left[\begin{array}{ccc} 0 & 0 & 0\\ 0 & 1 & 0\\ 0 & 0 & 1 \end{array}\right]$$

Let S⊆V be any surface, and area of orthogonal projections of rotated S is defined here as

(20) $$\begin{array}{cc} A_{P} & =Area\{x^{\prime }|x\;\in \;S\wedge x^{\prime }=P_{nk}\;R\left(\gamma ,\beta ,\alpha \right)x\}\\ & \equiv {O_{A\left(u_{n}^{\prime },u_{k}^{\prime }\right)}}_{\left\{n,k\right\}\in \left\{x,y,z\right\},n\neq k} \end{array}$$

where $O_{A(u_{n}^{\prime },{u^{\prime }}_{k})}$ stands for a generalized ‘orthogonal area operator’ on the appropriate semiaxis vectors 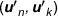 to obtain the area of the desired projection, and R(γ,β,α)∈SO(3) is presented here in Euler angles. Hence, the remaining task is to define the semiaxis vectors on the orthogonal projections and the corresponding $O_{A(u_{n}^{\prime },u_{k}^{\prime })}$. Finally, the constraints $c_{1-3}$ ensure that the total overlapping (obscured surface) area equals to $A_{P}$ for all projections.

#### 2.4.1 General ellipsoid and area projection

The area operator $O_{A(u_{n}^{\prime },u_{k}^{\prime })}$, defined in (20), for an ellipsoidal surface $S_{ell}\subseteq R^{3}$ is simply $|u_{n}^{\prime }\left|\right|u_{k}^{\prime }|\pi$, where $u_{\left\{n,k\right\}}^{\prime }$ are the semiaxis vectors for $x^{\prime }\in S^{\prime }$ that is, the projected shape on the static orthogonal planes. There are various ways finding $u_{\left\{n,k\right\}}^{\prime }$, but here Singular Value Decomposition (SVD) is utilized to solve the eigenvector directions and magnitudes, for which the latter relates inversely to the projected shape semiaxis lengths. Furthermore, SVD is a convenient method in obtaining the eigenvalues and the transformed basis particularly for an ellipsoidal shape, since an analogous operation is embedded within the visual interpretation of the transform itself.^24^ SVD states that for any m×n matrix,

(21) $$M=U\Sigma V^{T},$$

where U is a left orthogonal matrix corresponding to the basis vectors of the transformed coordinates, Σ holds the singular values of M, and $V^{T}$ is the transpose of the transformation from the static basis to the output domain. In another words, any matrix can be represented as three linear transformations corresponding to rotation, scaling and second rotation, with or without reflection. The quadratic matrix form of an ellipsoid is

(22) $$x^{T}\mathrm{Mx}=\left[x\;y\;z\right]\left[\begin{array}{ccc} \frac{1}{a^{2}} & 0 & 0\\ 0 & \frac{1}{b^{2}} & 0\\ 0 & 0 & \frac{1}{c^{2}} \end{array}\right]\left[\begin{array}{c} x\\ y\\ z \end{array}\right]=1,$$

where M is a ‘shape matrix’. The above formulation defines the ellipsoid surface in a cartesian frame aligned with its principal axes. However, the interest here lies in the ellipsoid in a rotated frame, or equivalently, in the rotated shape in the original coordinates. Rewriting (22) by applying rotation to the coordinates $y=\mathrm{Rx}\to x=R^{T}y$ yields

(23) $${\left(R^{T}y\right)}^{T}M\left(R^{T}y\right)=y^{T}\;\mathrm{RM}R^{T}y=y^{T}\;M^{\prime }y.$$

The equivalence is important so that SVD can be performed on the rotated shape matrix $M^{\prime }$. Applying projection and dimension erasing, and solving the singular values from $\Sigma (\sigma_{11},\sigma_{22})$ via SVD, expression in (20) becomes

(24) $$A_{P}=\frac{\pi }{\sqrt{\sigma_{11}\sigma_{22}}}$$

where subindices 11 and 22 correspond to the sorted (descending) magnitudes of σ, and $\sqrt{\sigma_{i}}$ are the eigenvalues of collapsed 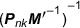. It is worth noting that for spheroids, only $\sigma_{22}$ is of interest, since $\frac{1}{\sqrt{\sigma_{11}}}=\left|u_{minor}\right|$ under all rotations.

#### 2.4.2 Ensuring SVD equivalence

A quantitative validation of the method is straightforward by demonstrating the correspondence of SVD with a numerical solution of a point cloud, enclosed by an ellipsoid surface

(25) $$\frac{x^{2}}{a^{2}}+\frac{y^{2}}{b^{2}}+\frac{z^{2}}{c^{2}}=1.$$

**FIGURE 2:**
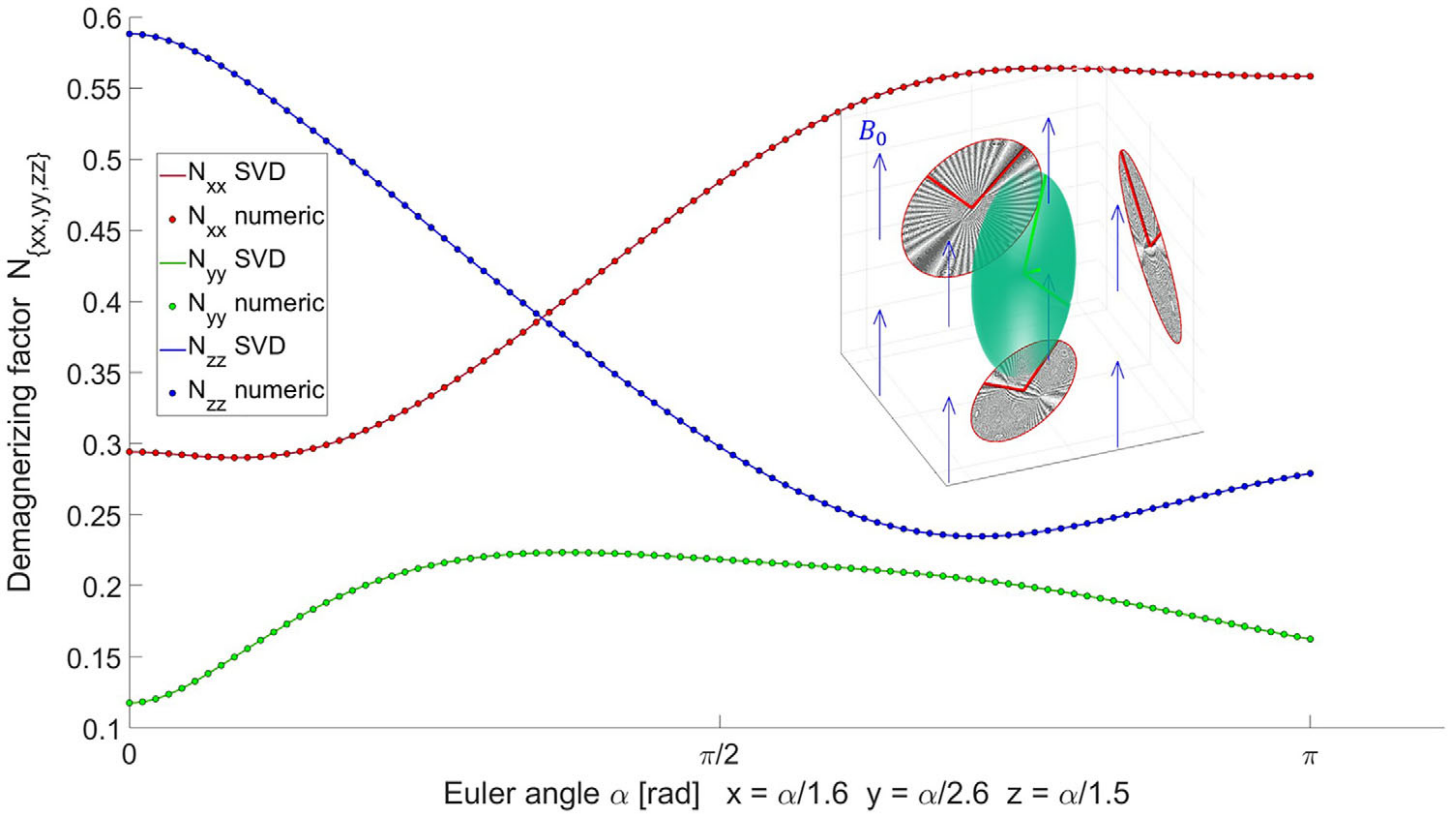
Example of demagnetizing factors of a general ellipsoid (a=2,b=5,c=1) under arbitrary rotations solved using Singular Value Decomposition (SVD) and via numerical evaluation of the convex hull area. The embedded figure provides a visual presentation of the numerical point‐cloud projections, shown as dark shaded areas. The corresponding SVD solutions are presented as red‐colored semiaxis vectors.

Finding the area of the projected convex hull on the orthogonal static planes after rotation yields the same result as with SVD, limited only by sampling and computational accuracy, presented in Figure 2.

The accuracy of the area‐projection approximation itself is most appropriately evaluated using the exact analytical solutions of (3)–(5), which can now be assessed conveniently by comparing the SVD approach and the direct tensor rotation.

## 3 Results

### 3.1 Model comparison: applicability of the area projection method

**FIGURE 3:**
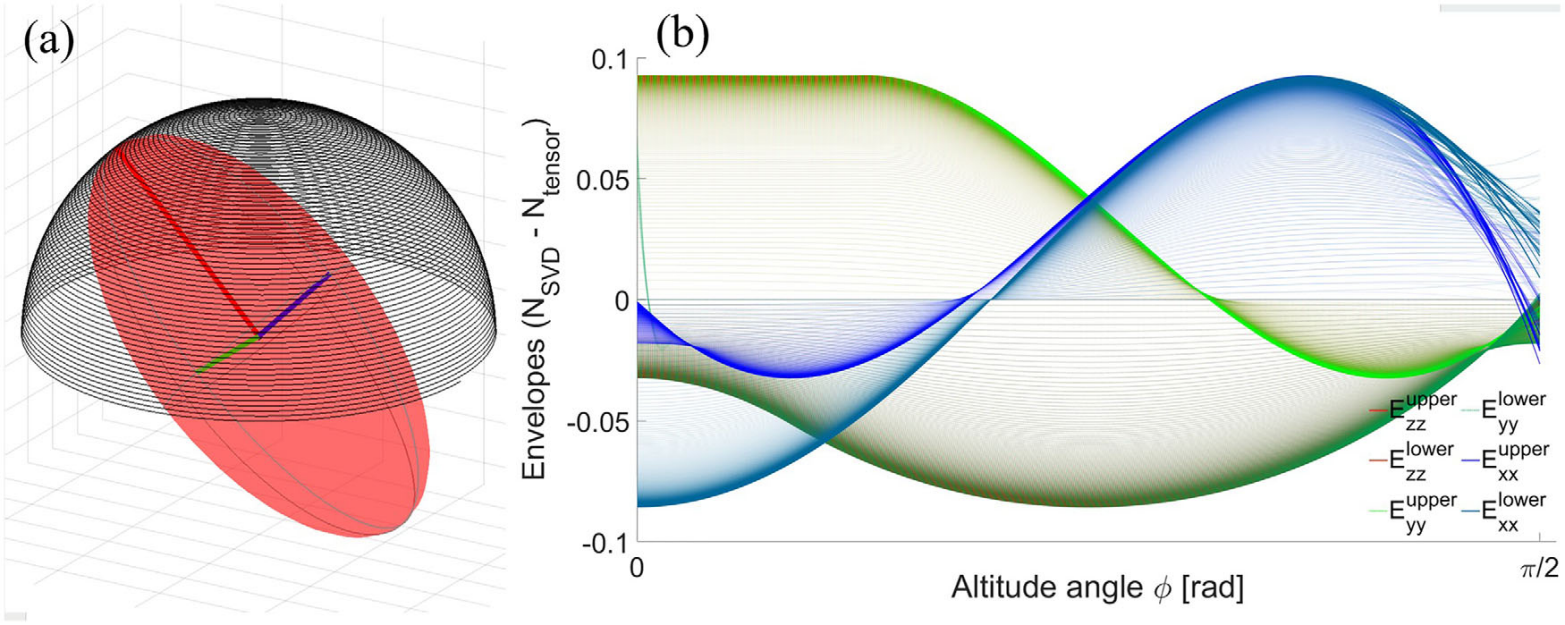
The area‐projection method using Singular Value Decomposition (SVD) is compared with directly rotating the demagnetizing tensor for prolate spheroids with various aspect ratios. The orthogonal solutions were computed using the expression in.^12^ The sampling function follows a hemispherical spiral path with a 0.005 rad ascent per revolution, illustrated in (a). The difference between the models is shown as upper and lower envelopes $E_{\mathrm{nn}}^{u,l}.$ Each envelope pair encloses an alternating curve (due to azimuthal rotation, not visualized) for different aspect ratios (b/a={0.999−0.0067},b=c). Higher line density corresponds to greater elongation of the spheroid.

The exact solutions for the orthogonal demagnetizing factors were utilized from ^12^ to construct a reference via direct tensor rotation. A quantitative analysis of the area‐projection approximation is presented in Figure 3.

**FIGURE 4:**
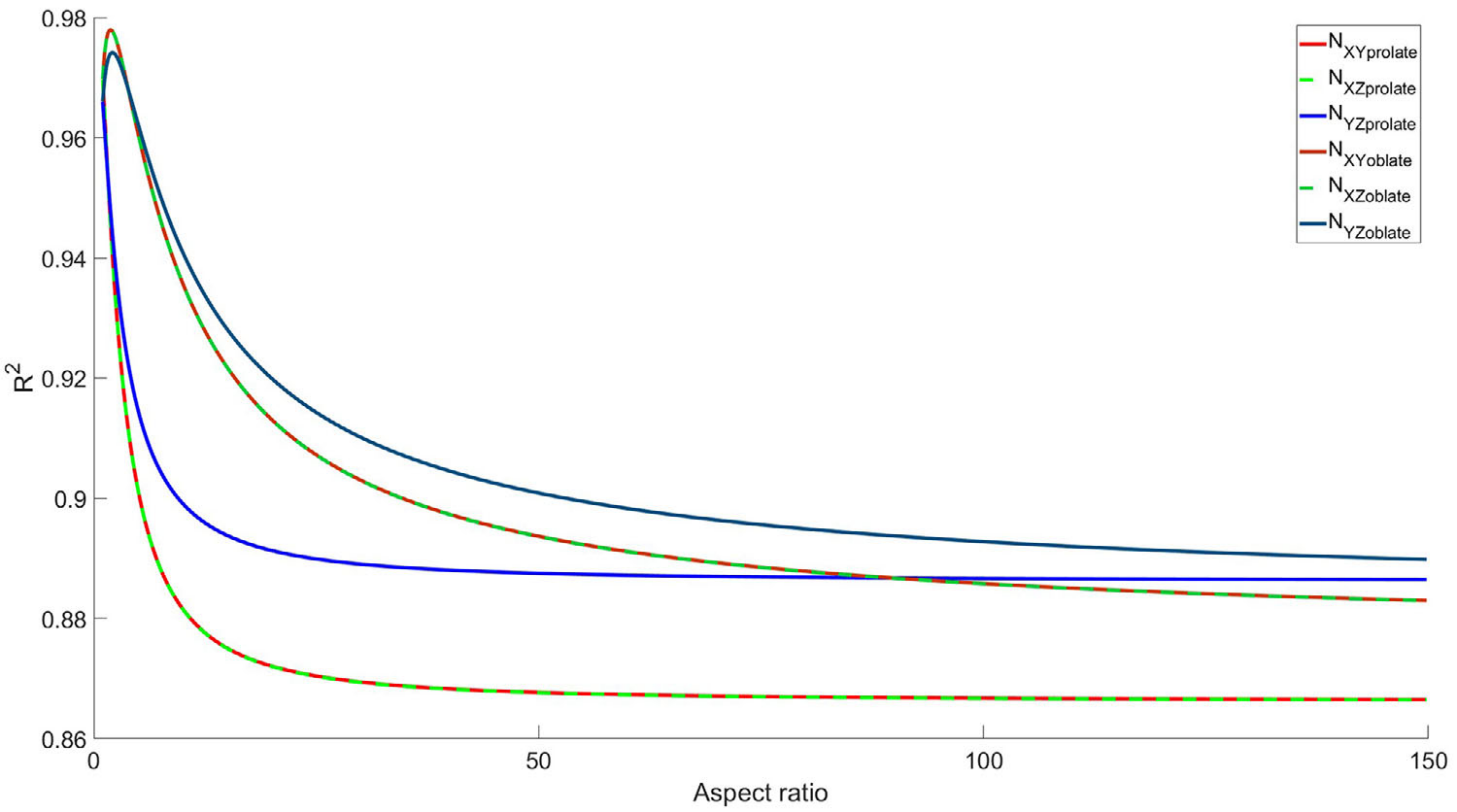
Model estimation of the SVD method in approximating the diagonal demagnetizing tensor components under rotations ($\theta =0-2\pi ,\varphi =0-\frac{\pi }{4}$) measured using the coefficient of determination $R^{2}$ as a function of aspect ratio.

Here each envelope corresponds to the maximum absolute error during azimuthal revolution for the given aspect ratio. Absolute error was chosen over relative error due to latter's inflated values, disproportionate to their physical significance when the inspected demagnetizing component is small, as also noted in.^9^ Finally, the model performance was evaluated as the function of aspect ratio over the sampled orientations via coefficient of determination $R^{2}$, presented in Figure 4.

The estimation in Figure 4 should not be interpreted as a definitive model performance measure, since it only quantifies how much of the diagonal component rotational variance is explained by the approximation. Note that Osborn's base formulas for prolate and oblate spheroids are undefined at a/b=1 due to a singularity. For the spherical case, the demagnetizing factors reduce to $N_{xx}=N_{yy}=N_{zz}=\frac{1}{3}$​, which must be treated separately. The disparity is apparent in Figure 4 as a/b→1, for which $R^{2}=1$ would be expected.

### 3.2 Translational force and torque before saturation magnetization

**FIGURE 5:**
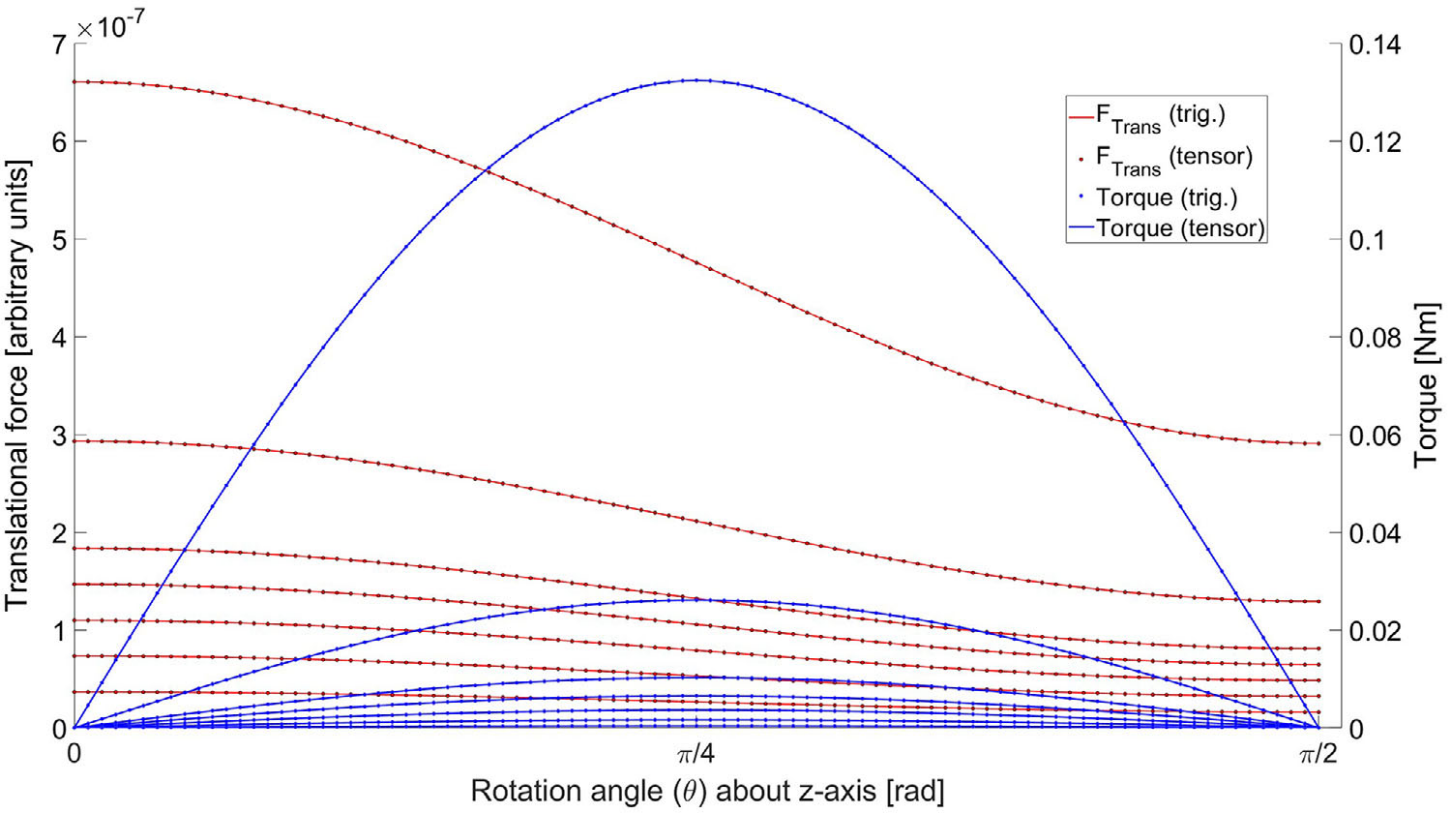
Translational force and torque at linear region. The tensor rotation is compared to the trigonometric scalar expressions in (7) and (10). Corresponding physical constants and field strengths were matched with.^16^ The force was computed by setting the field gradient strength to unity.

As shown in (14) and (15), the direct consequence of the tensor rotation generalization is the reformulation of the scalar expressions in (7) and (10). A comparison of the tensor rotation with respect to the standard scalar formulation is presented in Figure 5.

The results show exact correspondence within computational accuracy for χ≫1, as expected.

### 3.3 Saturation field magnitude

**FIGURE 6:**
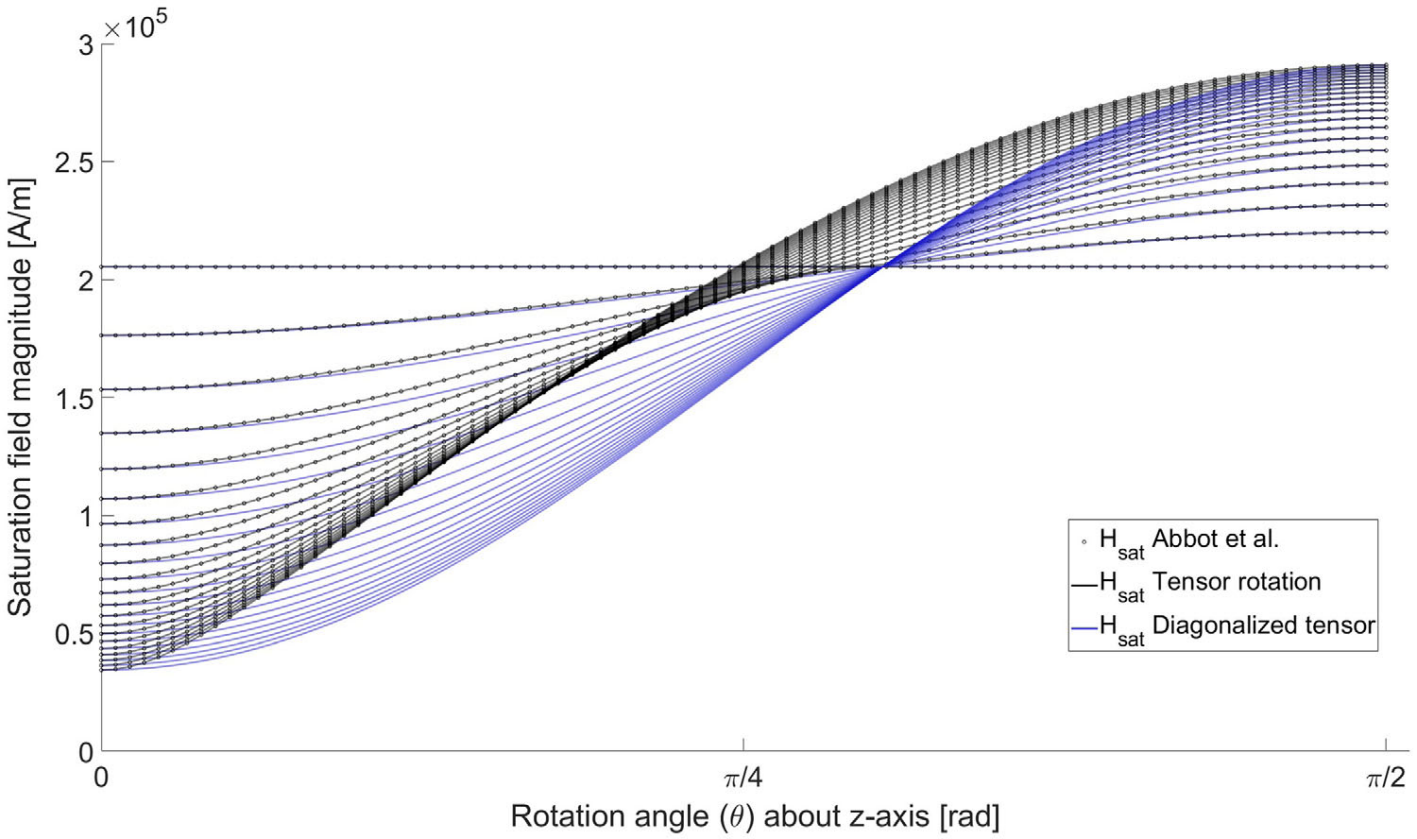
Saturation field strength for prolate spheroids. The results are computed using the tensor rotation method and compared with the expression in Abbott et al,^16^ with an exact match within computational accuracy. In addition, diagonal tensor component solutions are presented, naturally deviating from the full tensor solutions. Note that the diagonal solutions converge to a single point at $0.955317\;\mathrm{rad}=54.7356^{\circ }$.

Since magnetization behavior is dependent on magnetization saturation, it is sensible to recompute the angle‐dependent saturation field magnitudes. The comparison was performed between the full tensor solutions, the expression in ^16^ and the diagonal tensor terms for several aspect ratios, presented in Figure 6.

An interesting observation in Figure 6 is the convergence of the diagonal tensor solutions to a single point across all aspect ratios at a rather interesting angle of $54.7356^{\circ }$, commonly known as the MRI magic angle ϑ. That is, at ϑ the diagonal tensor components become completely independent of aspect ratio. Or in other words, the saturation field strength dependence on aspect ratio is solely due to off‐diagonal contribution.

### 3.4 Torque at saturation

**FIGURE 7:**
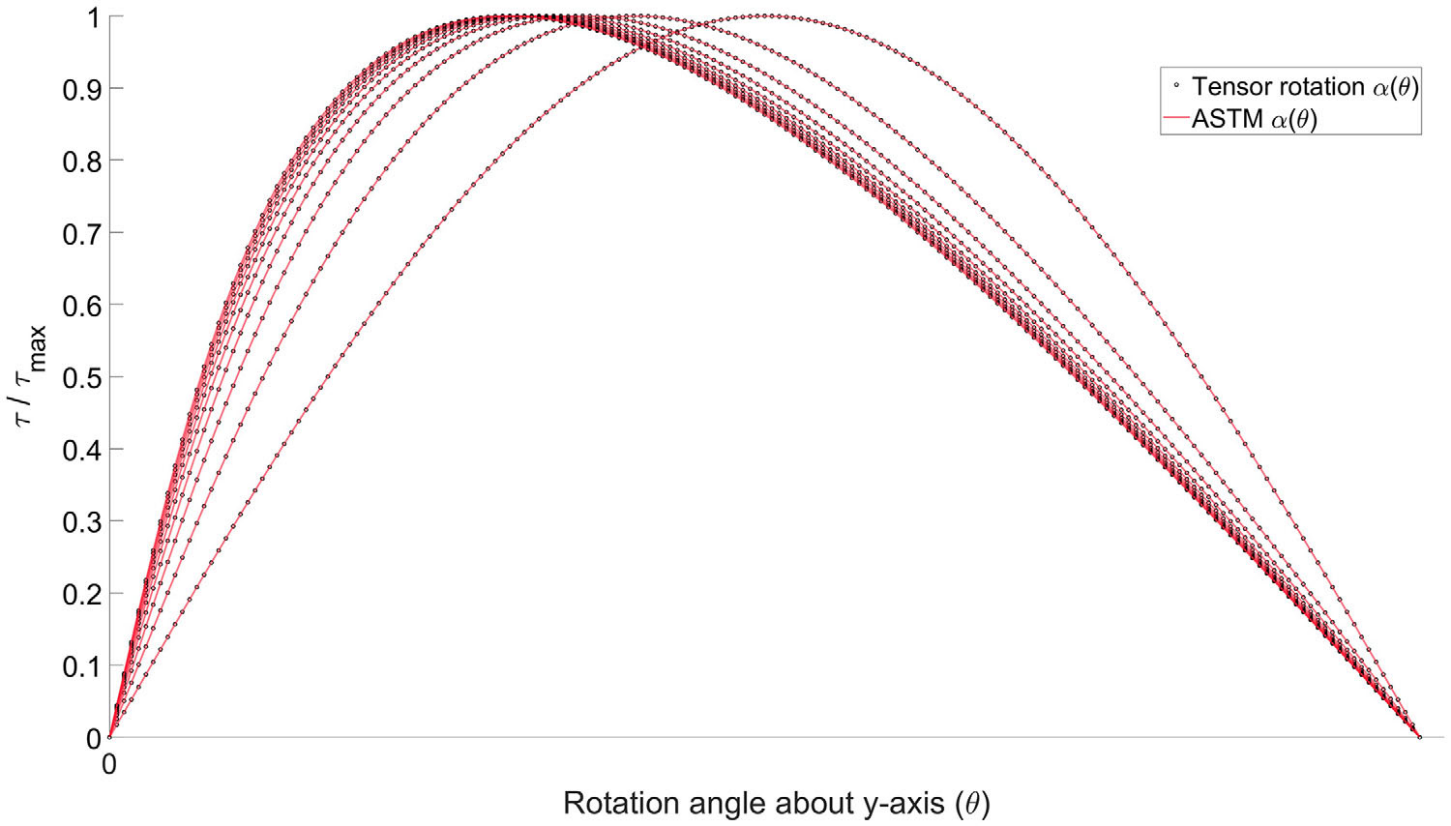
Example on the correspondence of normalized torque ($\tau /\tau_{max}$) between tensor rotation formulation and the ASTM standard ^17^ at saturation magnetization for few spheroidal aspect ratios $\frac{a}{b}=\left\{1-10\right\}$. The magnetization vector direction α(θ) was solved using the standard energy minimization approach. The expressions are equivalent when rotating the demagnetizing tensor with α(θ).

At saturation magnetization threshold, the magnitude of the magnetization vector maximizes, and the material can only respond to external field changes by angular dependence. Hence, calculating torque at saturation requires solving the nonlinearly dependent direction of $M_{s}$. Finding the roots for the scalar case in (11) and rotating the demagnetizing tensor with the resulting α instead of θ, yields an exact match with the ASTM formulation when plugged in (15), as expected. The comparison is presented in Figure 7.

The minimization procedure can be carried out equivalently in 3D via energy minimization in (16).

## 4 Discussion And Conclusions

Demagnetizing tensor determination and its practical approximations have important applications in magnet manufacturing, computational modeling, magnetic manipulation, and most notably, in magnetic resonance imaging. Particularly in medical contexts where magnetic safety is a major concern. The presence of foreign objects within the body is a common contraindication for MRI, primarily due to the induced eddy currents and heating, but also due to the translational forces and torque exerted by the strong magnetic field. This work provides means to further approximate the latter two under arbitrary orientations with minimal effort.

The main contribution of this work can be summarized as an examination of the effects of object rotation on demagnetizing tensors with respect to:

1. Generalized solutions for uniformly magnetized general ellipsoids obtained through simple tensor rotation.
2. Area‐projection approximation^9^ and its applicability under arbitrary rotations.
3. A generalized tensor‐based formulation for translational force and torque calculations.
4. Connecting diagonal components of the demagnetizing tensor and the corresponding saturation magnetic field magnitude to the well‐known magic angle.

The first point is a direct consequence of the geometric interpretation of demagnetizing tensors, simplifying the process of finding an exact solution under rotations, provided that a single base solution is known. The approach appears to be largely unnoticed in MRI research, although the presented formulation aligns with similar ideas in other contexts. These include early theoretical work by Moskowitz et al. ^20^ and recent applications in quantum magneto mechanics.^19^ The method is applied in generalized force, torque and saturation field calculations, thus leading in point 3 and 4.

The tensor formulation for the induced force and torque calculations showed an exact match for spheroidal rotations with ^16^ in the linear region. Equivalent results were found when considering saturation field strength in Figure 6. This is not surprising, since the derivation is analogous to its trigonometric version, merely generalizing the computation. The procedure can be carried out with any model incorporating exact solutions for orthogonal demagnetizing factors, as shown with the ASTM example in Section 3.4. The method is however limited at magnetization saturation, since finding the $M_{S}$ orientation requires a solution to the energy minimization problem in (16). This is straightforward numerically, but as such, does not fully support the rationale underlying this work. Alternative approaches should be inspected in future research.

The manifestation of the magic angle in Figure 6 as the converging point of prolate spheroid aspect ratios is also an interesting observation. Effectively, the diagonal demagnetizing factors become independent of the aspect ratio at this very specific orientation. The magic angle ϑ is traditionally derived from average dipolar interactions, formally described via a second order Legendre polynomial, for which the root is $arccos\;\left(\frac{1}{\sqrt{3}}\right)=54.7356^{\circ },$ corresponding to the convergence point under tensor rotation. The connection, however, only shows with the diagonal terms. At first glance, this observation appears solely as a mathematical property.

In point 2, a general non‐applicability of the area‐projection method is evident when considering any geometry not subject to $c_{1-3}$ in (18). For example, an arbitrary projection of a concave three‐dimensional surface does not ensure 1:1 linear mapping on to the obscured segment. Even for the applicable cases, the major pitfall lies in the definition of $O_{A(u_{n}^{\prime },u_{k}^{\prime })}$, which must be constructed individually for each geometric shape. Furthermore, only few geometries exhibit spatially uniform magnetization, which further restricts the direct applicability of the method. For instance, in rectangular prisms a single demagnetizing tensor cannot accurately represent the whole geometry prior full saturation, particularly near the vertices and non‐differentiable edges. This effectively leaves ellipsoids as the only geometry for which uniform magnetization can always be assumed. However, once a formal connection via (20) is constructed and underlying approximations are accepted, the method is applicable for all aspect ratios. In addition, SVD proves to be useful for the case of general ellipsoid due to its quadratic matrix form, but only when the transformed basis vector directions are of interest. Otherwise, simpler closed form expressions may be more suitable. Since non‐quadratic shapes do not have explicit single‐matrix definitions in the required form, one might consider computing an equivalent ellipsoid,^25^ solving the problem via trigonometry, or finding a solution to the point cloud similar to Figure 1. The first suggestion would be particularly interesting, as fast approximations using SVD could be constructed for arbitrary geometries and will be considered as a future aim.

The comparative results suggest that the area‐projection method is applicable for approximating the diagonal demagnetizing factors in spheroids with fair accuracy ($0.86<R^{2}<0.97$), comparable to the orthogonal cases. One should note that $R^{2}$ alone may not be sufficient for definite model estimation, and the absolute maximum errors should be also considered. In Figure 3, the absolute error increases with longer elongation, and becomes significant if the corresponding demagnetizing component is small. Although the present analysis is limited to quantitative results for prolate and oblate spheroids with finite sampling, the presented method and equations are constructed for a general ellipsoid and are straightforward to reproduce for independent validation.

It may be concluded that, for uniformly magnetized ellipsoids with a known base solution, one should consider directly transforming the corresponding demagnetizing tensor due to its computational simplicity. Moreover, approximations derived directly from axial lengths ^2^, ^9^ may not translate accurately to irregular geometries, not subject to $c_{1-3}$. In such cases, numerical solutions remain preferable, though the topic does carry some academic appeal. Accordingly, the method's practical value lies primarily in shapes that meet or closely approximate these conditions.

Finally, the practical utility of the generalized tensor rotation and the inspected approximative methods have potential in medical contexts. In the future, they might be utilized in developing and support imaging safety testing and evaluation, for example in medical device and implant manufacturing. It is important to note that the presented contributions are largely technical. In its current form, direct clinical use cases are limited and are not endorsed without further development and validation. In future, on‐site worst‐case scenario mapping of foreign bodies may be a potential developmental direction but for now, clinical applications remain as a long‐term motivation. Other potential applications lie in clinical education and basic research, in which the presented framework can be built upon. Future research and further validation will be required for any clinical implementation.

## FUNDING INFORMATION

The author has nothing to report.

## CONFLICT OF INTEREST STATEMENT

The author declares no conflicts of interest.
